# Psychological predictors of intention and avoidance of attending organized mammography screening in Norway: applying the Extended Parallel Process Model

**DOI:** 10.1186/s12905-021-01201-y

**Published:** 2021-02-15

**Authors:** Anna Ivanova, Ingela Lundin Kvalem

**Affiliations:** Department of Psychology, University of Oslo, Blindern, PB 1094, 0317 Oslo, Norway

**Keywords:** Breast cancer screening, Mammography, Extended parallel process model, Psychological predictors, Defensive avoidance, Intention

## Abstract

**Background:**

Mammography screening is the main method for early detection of breast cancer in Norway. Few studies have focused on psychological determinants of both attendance and non-attendance of publicly available mammography screening programs. The aim of the current study, guided by the Extended Parallel Process Model, was to examine how psychological factors influence defensive avoidance of breast cancer screening and intention to attend mammography.

**Methods:**

Cross-sectional survey data from a community sample of women living in Norway aged ≥ 18 (N = 270), and without a history of breast cancer, was collected from September 2018 to June 2019 and used to investigate the relationships between the Extended Parallel Process Model (EPPM) constructs and two outcomes: defensive avoidance of breast cancer screening and intention to attend mammography within the next two years. After adjusting for confounding factors, the hierarchical multiple linear regression analyses was conducted to assess the ability of the independent variables based on the EPPM to predict the two outcome variables. Significance level was chosen at *p* < 0.05.

**Results:**

Multivariate analyses showed that defensive avoidance of breast cancer screening was predicted by lower perceived susceptibility to breast cancer (β =  − 0.22, *p* = 0.001), lower response efficacy of mammography screening (β =  − 0.33, *p* = 0.001), higher breast cancer fear (β = 0.15, *p* = 0.014), and checking breasts for lumps (β =  − 0.23, *p* = 0.001). Intention to attend mammography within the next two years was predicted by higher response efficacy of mammography screening (β = 0.13, *p* = 0.032), having a lower educational level (β =  − 0.10, *p* = 0.041), and regular previous mammography attendance compared to never attending (β = 0.49, *p* = 0.001).

**Conclusions:**

The study revealed that defensive avoidance of breast cancer screening and intention to attend mammography were not predicted by the same pattern of psychological factors. Our findings suggest future health promotion campaigns need to focus not only on the psychological factors that encourage women’s decision to attend the screening, but also to counter factors that contribute to women’s decision to avoid it.

## Background

Breast cancer is the most common type of cancer among women both in developed and developing countries [1]. Breast cancer is currently the second leading cause of cancer deaths among women in Norway with an increase in survival rates from 73.7% in 1980 to 92.0% in 2019. This can be attributed to new methods of treatment, as well as a national screening program [2], where mammography is the main method [3].

The Norwegian Breast Cancer Screening Program (NBCSP) is free (apart from a user fee of 250 NOK ($25)) and invites all women aged 50–69 years to attend mammography screening every second year. Women with familial risks of breast cancer are also invited to attend screening annually between the age of 30 and 60, and every second year after that [4]. A recent evaluation of NBCSP concluded that the program has contributed to 20–30% reduction in breast cancer mortality for women between 50 and 79 years [4]. The impact of breast cancer screening is also named as one of the reasons for slightly higher rates of long-term survival in the 50–59 age group compared to the below 50 age group [2].

The Norwegian nationwide mammography participation rate is ca. 76%, however, in some parts of the country (e.g. Oslo county) rates are below the 70% recommended by the European guidelines [5, 6]. Lagerlund, Sparén [7] note that in the countries with publicly available screening programs and high attendance rates, understanding why women do not attend the screening may help to better reach non-attending groups.

Few studies have examined factors that may contribute to both attendance and non-attendance of mammography screening when it is free, as in Norway [6–15]. Previous research has addressed several sociodemographic factors (e.g. age, ethnicity, residence) [6, 10–13] and system-related issues (e.g. trust in the healthcare system, receiving an invitation to screening and routinization of mammography screening) [14, 15]. However, the majority of the studies examining psychological predictors of breast cancer screening focus on determinants of screening attendance, rather than non-attendance [7]. This paper presents a study that offers theory-based investigation of psychological predictors of mammography screening.

The Extended Parallel Process Model (EPPM) was used as a conceptual framework to structure the variables and analysis in this study. While not as widely used as other social cognition models, the EPPM has been previously applied to study a variety of health behaviors [16]. It has also shown some promising results in a cancer prevention research on skin cancer prevention [17], colorectal cancer screening [18], testicular self-examination [19, 20], HPV prevention [21], and clinical breast examination [22]. The model attempts to explain both adaptive and maladaptive responses to a health threat and incorporates constructs that have been previously associated with breast screening behaviors: perceived susceptibility [23–25], perceived severity [26], response efficacy [27], self-efficacy [28–30], and fear [8, 31, 32]. The EPPM distinguishes between two processes—danger control and fear control [33]. According to the EPPM, when perceived threat (comprised of perceived severity of health threat and perceived susceptibility to health threat) is high, and perceived efficacy (comprised of response efficacy and self-efficacy) is high, a person would engage in danger control processes, predominantly cognitive processes, which leads to an adaptive response (e.g. positive behavioral change) [33, 34]. However, if perceived threat is high but perceived efficacy is low, fear of threat becomes more intense and a person would engage in fear control, largely emotional processes, which result in maladaptive response, for example defensive avoidance or reactance [33, 34]. In the EPPM, fear (a negative emotional reaction elicited by a perceived threat) becomes one of the central components of the model and is considered to cause maladaptive behavior [33]. The EPPM further posits that perceived threat and perceived efficacy may be influenced by individual differences which could increase the probability of maladaptive responses [33].

**Fig. 1:**
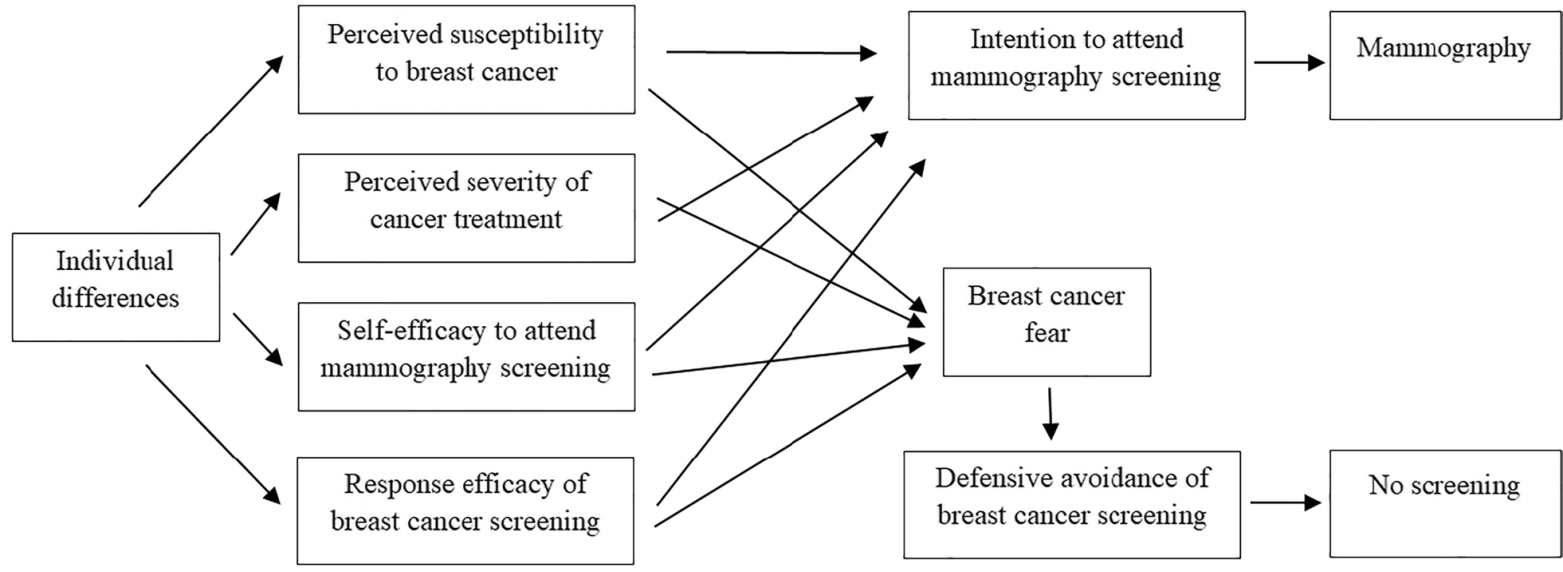
Adapted version of the Extended Parallel Process Model to predict mammography screening in the current study

The model was adapted for the current study based on the cross-sectional study design and prior cancer-related research (see Fig. 1). Due to generally high perceptions of cancer severity, previous studies found little variance in cancer severity scores [18, 35]. Champion [35] suggests using perceived susceptibility alone to measure perceived threat of breast cancer. Following Champion [35], perceived susceptibility to breast cancer was chosen as a measure of perceived threat variable. Separately, perceived severity of cancer treatment was included as it was predictive of mammography attendance in prior research [8]. To narrow down the outcome measures for adaptive response, the study focuses on intention to attend mammography screening. Maladaptive response was conceptualized as defensive avoidance of breast cancer screening.

The main objective of the present study was to examine which psychological factors are associated with intention to attend mammography and defensive avoidance of breast cancer screening in the Norwegian context. In accordance with EPPM, it was hypothesized that defensive avoidance of breast cancer screening would be positively associated with breast cancer fear and negatively associated with perceived efficacy and perceived threat, while intention to attend mammography screening would be positively related to perceived threat and perceived efficacy.

## Methods

### Design and sampling

The cross-sectional study was conducted as an addition to The Breast Size Satisfaction Survey (BSSS) [36]. A convenience sample consisting of 296 adult (≥ 18 years old) women living in Norway completed a self-administered paper and pencil questionnaire between September 2018 and June 2019. All methods were carried out in accordance with relevant guidelines and regulations. The study was in accordance with Helsinki declaration, and ethical clearance was granted by the Research Ethics Committee at the Department of Psychology, University of Oslo. Participants were presented with information about the research project on a separate information sheet and on the front page of the questionnaire. On the first page they were informed that they consented to take part in the study by completing and returning the anonymous questionnaire. The participants were recruited from the community using direct approaches in different sites of congregate activity, such as at mammography centers and public locations in Oslo at different times of the day. To include more women from different ethnic backgrounds questionnaires were also distributed through women support organizations and churches, as well as through snowball sampling. To avoid selection bias and extend the sampling outside the urban area, this method was supplemented with telephone registry random sampling. The overall response rate was ca. 30%. The final sample consisted of 270 adult women with no history of breast cancer. We excluded 26 participants: 14 responses were not completed enough to be used in the analysis and 12 women reported prior breast cancer diagnosis.

### Instrument

The questionnaire used in the study (see Additional file 1) consisted of two parts: the first part was provided by the BSSS project [36]; the second part was developed by the authors based on the literature review of breast cancer screening research. Construct validity was assessed using factor analysis with orthogonal rotation. The reliability of the scales was examined using Cronbach’s alpha and Spearman-Brown coefficient (for the scales that contained two items). Information leaflets and questionnaires were available in Norwegian and English. The questionnaire was originally prepared in English and then translated into Norwegian. It was then back-translated into English by an independent translator.

### Measures

#### Dependent variables

*Intention to attend mammography in the next two years* Intention to attend mammography screening and was measured with a single statement “I intend to attend mammography in the next two years” with four response options from *No* (1) to *Yes, definitely* (4).

*Defensive avoidance of breast cancer screening* Based on prior research [37, 38], defensive avoidance was measured using five beliefs statements (e.g. “I do not have any symptoms so I don’t need to do a mammogram”, “My health is too good at the moment to worry about breast cancer”) with five response categories from “*Totally disagree* (1)” to “*Totally agree* (5)”. A high mean score indicates high defensive avoidance. The scale had good internal consistency (α = 0.73).

#### Independent variables

*Demographic variables* included age, ethnicity, place of residence, education level and financial security.

*Breast screening behaviors* included breast checks frequency and prior mammography attendance. Breast checks frequency that was assessed with one item “How often do you check your breast?”, rated from “*Rarely or never* (1)” to “*At least once a week* (4)” [36]. Participants were later coded into two groups: *rarely or never* and *weekly to yearly*. Mammography attendance was measured using two items: a yes/no question worded as “Have you ever had a mammogram?”, and a question about mammography frequency: “How often do you go to get a mammogram?” with response options “*Never* (1)”, “*Only if I get symptoms*”, “*Less than once in two years*”, “ *Once in two years*”, “*Once a year* (5)”. Mammography frequency was coded into three groups: *never, irregular* and *regular*. One item asked about the use of NBCSP: “How did you get a mammogram the last time?” with response options “*Through the national screening program* (1)” and “*My own initiative* (2)”.

#### EPPM components

*Perceived susceptibility* Based on the scale by Gurmankin Levy, Shea [39], perceived susceptibility was assessed using two items: “I believe my chance of developing breast cancer in my lifetime is…”, rated from “*Very low* (1)” to “*Very high* (5)”, and “My chance of developing breast cancer compared to the average woman of my age is…”, rated from “*Much lower* (1)” to “*Much higher* (5)”. The scale was reliable (Spearman-Brown coefficient = 0.81).

*Perceived severity of cancer treatment* was assessed with a single item [8]: “I believe that breast cancer treatment is hard to endure”, with 5 response categories ranging from “*Totally disagree* (1)” to “*Totally agree* (5)”.

*Perceived efficacy* Self-efficacy was operationalized as specific self-efficacy—a belief that a person can overcome difficulties to go through with mammography screening [40] and was measured with three questions based on items from the mammography barriers scale [37]: “I am certain I can attend mammography even though I have little time”, “I am certain I can attend mammography even though I am embarrassed to expose my body to others”, and “I am certain I can attend mammography even though mammography may be painful”. Response options ranged from “*Totally disagree* (1)” to “*Totally agree* (5)”. The items were included in the analysis separately.

*Response efficacy* was operationalized as a degree to which a person believes taking mammography is beneficial for them. Based on the scale by Champion [35, 41], response efficacy was measured with four items indicating beneficial effects of taking mammography (e.g. “Getting a mammogram would make me feel safe”), rated from “*Totally disagree* (1)” to “*Totally agree* (5)”. The scale had good internal consistency (α = 0.72).

*Breast cancer fear.* Three items adapted from the scales by Lerman, Track [42] and Champion [43] were used to assess different aspects of breast cancer-related fear (e.g. “I worry a lot about developing breast cancer”), rated from *Totally disagree* (1) to *Totally agree* (5). The internal consistency of the scale was slightly low (α = 0.69).

*Individual differences* in trait anxiety, self-esteem and perceived health, were assessed with items from the BSSS [36]. Trait anxiety was measured with a single item “I see myself as anxious and easily upset” with five response options from “*Totally disagree* (1) to “*Totally agree* (5)”. Self-esteem was measured using a single item represented by a statement “I have high self-esteem” with seven response options from “*Not very true of me* (1)” to “*Very true of me* (7)”. Perceived health was assessed by a single item frequently used in previous research [44] “For someone my age my health is generally…” that had five response options: *bad* (1)/*not good/ordinary/good/excellent* (5).

### Statistical analyses

The data was analyzed with IBM SPSS Statistics version 25. Descriptive statistics were generated to describe the study sample and examine distribution of scores. We obtained means with standard deviations for continuous variables and proportions for categorical variables. A series of t-tests (for such variables as place of residence, ethnicity, breast checks frequency and education) and One-way analysis of variance (ANOVA) tests (for financial security and mammography frequency) were performed to compare mean values of intention and defensive avoidance between groups of categorical predictors. A series of correlations were performed to examine relationships between continuous variables (age, trait anxiety, self-esteem, perceived health, perceived susceptibility to breast cancer, perceived severity of cancer treatment, three self-efficacy variables, response efficacy of mammography screening, and breast cancer fear) and each of the two dependent variables (intention and defensive avoidance). Hierarchical multiple linear regression analyses were conducted to assess the ability of the EPPM-related independent variables (perceived susceptibility to breast cancer, perceived severity of cancer treatment, three self-efficacy measures, response efficacy of mammography screening, and breast cancer fear) to predict defensive avoidance of breast cancer screening and intention to attend mammography, after controlling for the influence of other potential predictors. For each of the outcome variables, hierarchical multiple linear regression analyses included three steps. Demographic variables that were significantly associated with each of the two outcome variables in the bivariate analyses were entered at step 1, EPPM model variables were included at step 2. We also tested how previous breast screening behaviors (breast checks frequency and mammography frequency) influenced intention to attend mammography. Considering that previous behavior is usually a strong predictor of the future behavior [45], their influence was examined in step 3. All analyses were done with bias corrected accelerated (BCa) bootstrapping. Significance level was chosen at *p* < 0.05.

## Results

The age of the participants ranged between 19 and 88 years old (*M* = 48.26; *SD* = 15.51). 79.0% of the participants were ethnic Norwegian, 21.0% had migration background. The majority of participants (72.2%) resided in urban areas and 63.7% reported having higher than secondary education. Approximately half (53.7%) of the participants reported feeling as financially secure as other people of their age, and more than one third (37.0%) reported feeling more financially secure than other people of their age. In our sample, 66.5% of participants (179 women) reported having attended mammography at least once in their life, among them 78.0% reported doing so through the mammography program.

Tables 1 and 2 show the results of bivariate analyses for categorical (Table 1) and continuous (Table 2) variables. For defensive avoidance of breast cancer screening, the bivariate analyses for categorical predictors showed that significantly higher mean scores in defensive avoidance were reported by women with migration background (*p* < 0.001), some form of higher education (*p* = 0.023), and those who rarely or never checked their breasts for lumps (*p* < 0.001). Women that reported regular mammography frequency scored significantly lower in defensive avoidance than women that reported attending mammography irregularly or never (*p* < 0.001). Age (Table 2) was weakly negatively correlated with defensive avoidance (*r* =  − 0.19, *p* = 0.004). Among the theoretical model variables, defensive avoidance was significantly positively associated with perceived severity of cancer treatment (*r* = 0.18, *p* = 0.006) and breast cancer fear (*r* = 0.20, *p* = 0.002), and significantly negative associated with perceived susceptibility to breast cancer (*r* =  − 0.31, *p* < 0.001), self-efficacy to attend mammography despite having little time (*r* =  − 0.38, *p* < 0.001), and response efficacy of mammography screening (*r* =  − 0.47, *p* < 0.001).

**Table 1:** T-tests and One-way ANOVA tests investigating differences in defensive avoidance and intention for potential categorical predictors

Defensive avoidance						Intention to attend mammography						
	T-test											
	n	M	SD	t	CIs^a^	n	M	SD		t		CIs^a^
Place of residence				1.40	[− 0.03; 0.30]					− 3.26**		[− 0.63; − 0.19]
Urban	195	1.77	0.66			194	2.99	0.97				
Rural	74	1.65	0.57			73	3.40	0.74				
Ethnicity				− 4.37***	[− 0.60; − 0.22]					0.05		[− 0.25; 0.27]
Ethnic Norwegian	211	1.65	0.60			209	3.10	0.94				
Migrant	56	2.06	0.68			56	3.09	0.90				
Breast checks frequency				5.34***	[0.25; 0.55]					− 3.00**		[− 0.55; − 0.13]
Rarely or never	122	1.96	0.66			121	2.91	0.89				
Weekly to yearly	147	1.55	0.57			146	3.25	0.93				
Education level				− 2.18*	[− 0.32; − 0.03]					3.68**		[0.21; 0.63]
High school or less	95	1.63	0.60			94	3.37	0.80				
Some form of higher education	172	1.80	0.66			171	2.95	0.95				

	One-way ANOVA									
	n	M	SD	F	Differences	n	M	SD	F	Differences
Financial security				1.25					0.20	
Less secure	24	1.93	0.69			24	3.00	1.02		
Same	145	1.71	0.61			143	3.13	0.91		
More secure	100	1.72	0.67			100	3.09	0.93		
Mammography frequency				13.35***	R < I^b^				82.62***	R > I^d^
Regular	136	1.55	0.58		R < N^c^	136	3.65	0.61		R > N^e^
Irregular	41	1.91	0.64			40	2.73	0.78		
Never	93	1.74	0.66			92	2.43	0.86		

R, regular; I, irregular; N, never

^a^BCa bootstrap 95% confidence intervals (CIs) for mean differences between the groups of categorical predictors

^b^Mean difference =  − 0.36, BCa bootstrap 95% CIs [− 0.59; − 0.15]

^c^Mean difference =  − 0.40, BCa bootstrap 95% CIs [− 0.56; − 0.24]

^d^Mean difference = 0.93, BCa bootstrap 95% CIs [0.65; 1.19]

^e^ Mean difference = 1.22, BCa bootstrap 95% CIs [1.01; 1.41]

*p < 0.05; **p < 0.01; ***p < 0.001

**Table 2:** Bivariate correlations between the study variables

	Variables	1	2	3	4	5	6	7	8	9	10	11	12	13
1	Defensive avoidance	1												
2	Intention	− 0.30*** [− 0.43; − 0.18]	1											
3	Age	− 0.19** [− 0.33; − 0.05]	0.49*** [0.37; 0.60]	1										
4	Trait anxiety	0.08 [− 0.05; 0.20]	− 0.14* [− 0.27; − 0.02]	− 0.24*** [− 0.37; − 0.11]	1									
5	Self-esteem	− 0.05 [− 0.19; 0.08]	− 0.05 [− 0.09; 0.19]	0.16* [0.03; 0.28]	− 0.42*** [− 0.54; − 0.31]	1								
6	Perceived health	0.02 [− 0.11; 0.16]	.02 [− 0.09; 0.14]	− 0.10 [− 0.21; 0.01]	− 0.20** [− 0.31; − 0.09]	0.25*** [0.15; 0.37]	1							
7	Perceived susceptibility	− 0.31*** [− 0.44; − 0.18]	0.02 [− 0.12; 0.16]	− 0.12 [− 0.27; 0.02]	0.17** [0.03; 0.30]	− 0.13* [− 0.27; 0.02]	− 0.11 [− 0.23; 0.002]	1						
8	Perceived severity of cancer treatment	0.18** [0.07; 0.29]	− 0.11 [− 0.25; 0.03]	− 0.19** [− 0.31; − 0.07]	0.24*** [0.12; 0.36]	− 0.16* [− 0.28; − 0. 02]	− 0.07 [− 0.19; 0.06]	0.04 [− 0.08; 0.16]	1					
9	Self-efficacy, time	− 0.38*** [− 0.49; − 0.25]	0.38*** [0.26; 0.50]	0.18** [0.03; 0.33]	0.001 [− 0.13; 0.12]	− 0.04 [− 0.14; 0.06]	0.07 [− 0.05; 0.18]	0.17** [0.03; 0.31]	− 0.12 [− 0.25; 0.01]	1				
10	Self-efficacy, embarrassment	− 0.06 [− 0.18; 0.06]	− 0.06 [− 0.18; 0.08]	− 0.23*** [− 0.35; − 0.11]	− 0.002 [− 0.13; 0.12]	− 0.05 [− 0.17; 0.08]	.005 [− 0.12; 0.13]	0.17** [0.05; 0.29]	0.03 [− 0.09; 0.16]	0.22*** [0.10; 0.35]	1			
11	Self-efficacy, pain	− 0.08 [− 0.19; 0.04]	− 0.06 [− 0.18; 0.08]	− 0.15* [− 0.27; − 0.01]	− 0.03 [− 0.16; 0.10]	− 0.03 [− 0.15; 0.10]	− 0.02 [− 0.15; 0.12]	0.20** [0.07; 0.31]	0.05 [− 0.08; 0.19]	0.20** [0.07; 0.32]	0.71*** [0.64; 0.79]	1		
12	Response efficacy	− 0.47*** [− 0.59; − 0.35]	0.30*** [0.17; 0.42]	0.06 [− 0.08; 0.21]	0.05 [− 0.09; 0.17]	0.01 [− 0.11; 0.13]	0.01 [− 0.13; 0.15]	0.12 [− 0.02; 0.25]	− 0.07 [− 0.20; 0.05]	0.45*** [0.30; 0.59]	0.08 [− 0.03; 0.18]	0.10 [− 0.02; 0.23]	1	
13	Breast cancer fear	0.20** [0.06; 0.31]	− 0.18** [− 0.31; − 0.04]	− 0.35*** [− 0.46; − 0.23]	0.34*** [0.24; 0.44]	− 0.17** [− 0.30; − 0.05]	− 0.19** [− 0.31; − 0.04]	0.16* [0.03; 0.29]	0.27*** [0.14; 0.39]	− 0.12 [− 0.26; 0.03]	0.14* [0.02; 0.27]	0.02 [− 0.11; 0.15]	− 0.13* [− 0.27; 0.01]	1

All correlation coefficients are Pearson *r*. BCa bootstrap 95*%* CIs reported in brackets

*p < 0.05; **p < 0.01; *** *p* < 0.001

For intention to attend mammography, the bivariate analyses for categorical predictors (Table 1) showed that intention to perform breast self-exam within the next month was significantly associated with the place of residence (*p* = 0.001), education level (*p* = 0.001), breast checks frequency (*p* = 0.003), and mammography frequency levels (*p* < 0.001). Intention was moderately positively correlated with age (*r* = 0.49, *p* < 0.001) and weakly negatively correlated with trait anxiety (*r* =  − 0.14, *p* = 0.028) (Table 2). Among the EPPM model variables, intention had significant positive associations with self-efficacy to attend mammography despite having little time (*r* = 0.38, *p* < 0.001) and response efficacy of mammography screening *(r* =  − 0.29, *p* < 0.001), and negative correlation with breast cancer fear (*r* =  − 0.18, *p* = 0.005). Variables that were statistically significant in the bivariate analysis as well as the EPPM model variables were subsequently included in the multiple hierarchical regression models. As in the EPPM fear has no direct relationship with intention, breast cancer fear was not included in the regression model to predict intention to attend mammography.

Table 3 shows a hierarchical multiple linear regression model to predict defensive avoidance. Step 1 of the hierarchical regression significantly predicted defensive avoidance explaining 8.7% of variance in defensive avoidance scores (*F* (3, 248) = 8.86, *p* < 0.001). At this step, ethnicity was the only significant predictor of defensive avoidance (β = 0.24, *p* = 0.002). With addition of the EPPM variables, the model explained 32.6% of the total variance in defensive avoidance (*F*(10, 238) = 13.02, *p* < 0.001). At this step, the significant unique predictors of defensive avoidance were response efficacy (β =  − 0.33, *p* = 0.001), perceived susceptibility (β =  − 0.25, *p* = 0.001), and breast cancer fear (β = 0.16, *p* = 0.014). Addition of breast checks frequency and mammography frequency significantly increased prediction and the final model accounted for 36.8% of variance in defensive avoidance (*F* (13, 235) = 12.09, *p* < 0.001). Perceived susceptibility, response efficacy, and breast cancer fear remained significant predictors of defensive avoidance, and higher breast checks frequency was significantly associated with defensive avoidance (β =  − 0.23, *p* = 0.001). There was no association between mammography frequency and defensive avoidance.

**Table 3:** Hierarchical multiple regression analysis predicting defensive avoidance of breast cancer screening

Entered variables	Adj. R^2^	F	B	SE B	β	*t*	*p*	95% BCa confidence intervals for B	
								Lower	Upper
**Model 1**	**0.09**	8.86***							
Age			− 0.01	0.00	− 0.12	− 1.83	0.116	− 0.01	0.00
Ethnicity			0.36	0.10	**0.24**	3.75	0.**002**	0.17	0.56
Education (HS: 0; HE:1)			0.11	0.08	0.08	1.26	0.195	− 0.07	0.26
**Model 2**	**0.33**	13.02***							
Age			− 0.00	0.00	− 0.08	− 1.39	0.272	− 0.01	0.00
Ethnicity			0.11	0.089	0.07	1.201	0.221	− 0.05	0.25
Education (HS: 0; HE:1)			0.06	0.07	0.05	0.87	0.376	− 0.09	0.20
Perceived severity of cancer treatment			0.06	0.04	0.09	1.61	0.095	− 0.01	0.13
Perceived susceptibility			− 0.22	0.06	− **0.25**	− 4.39	**0.001**	− 0.33	− 0.11
Self-efficacy despite lack of time			− 0.06	0.05	− 0.10	− 1.59	0.259	− 0.17	0.04
Self-efficacy despite embarrassment			− 0.02	0.03	− 0.05	− 0.60	0.567	− 0.08	0.05
Self-efficacy despite pain			0.03	0.04	0.05	0.70	0.476	− 0.05	0.09
Response efficacy			− 0.39	0.08	− **0.33**	− 5.56	**0.001**	− 0.55	− 0.24
Breast cancer fear			0.11	0.05	**0.16**	2.67	**0.014**	0.03	0.20
**Model 3**	**0.37**	12.09***							
Age			− 0.00	0.00	− 0.02	− 0.22	0.878	− 0.01	0.01
Ethnicity			0.08	0.09	0.05	0.93	0.347	− 0.08	0.23
Education (HS: 0; HE:1)			0.09	0.07	0.06	1.18	0.220	− 0.06	0.21
Perceived severity of cancer treatment			0.04	0.04	0.06	1.10	0.268	− 0.03	0.11
Perceived susceptibility			− 0.20	0.06	− **0.22**	− 3.95	**0.001**	− 0.31	− 0.08
Self-efficacy despite lack of time			− 0.03	0.05	− 0.04	− 0.64	0.619	− 0.13	0.07
Self-efficacy despite embarrassment			− 0.02	0.03	− 0.04	− 0.55	0.577	− 0.08	0.05
Self-efficacy despite pain			0.03	0.04	0.07	0.92	0.368	− 0.03	0.09
Response efficacy			− 0.38	0.08	− **0.33**	− 5.65	**0.001**	− 0.53	− 0.25
Breast cancer fear			0.11	0.04	**0.15**	2.70	**0.014**	0.03	0.19
Breast checks frequency (Never: 0; W/Y: 1)			− 0.30	0.08	− **0.23**	− 4.22	**0.001**	− 0.44	− 0.15
Mammography frequency Never 0 versus Irregular 1			− 0.06	0.13	− 0.05	− 0.59	0.618	− 0.33	0.20
Never 0 versus Regular 1			− 0.02	0.13	− 0.01	− 0.16	0.896	− 0.26	0.24

Results based on 1000 samples BCa bootstrapping

Bold values indicate statistically significant results (*p* < 0.05)

HS, high school and less; HE, some form of higher education; W/Y, weekly to yearly

***p < 0.001

Table 4 shows the results of a hierarchical multiple linear regression analysis to predict intention to attend mammography. Step 1 significantly predicted intention to attend mammography explaining 25.7% of variance in intention scores (*F* (4, 241) = 22.16, *p* < 0.001). At this step, age (β = 0.43, *p* = 0.001) and education level (β =  − 0.16, *p* = 0.007) were significant unique predictors of intention. With addition of EPPM variables, the model accounted for additional 11.3% of the variance in intention to attend mammography (explaining 35.6% of the total variance in intention (*F* (10, 235) = 14.55, *p* < 0.001). Age and education remained as unique predictors of intention to attend mammography. Among the EPPM constructs, significant predictors of intention were self-efficacy to attend mammography despite having little time (β = 0.24, *p* = 0.006) and response efficacy of mammography screening (β =  − 0.16, *p* = 0.012).

**Table 4:** Hierarchical multiple regression analysis predicting intention to attend mammography

Entered variables	Adj. R^2^	F	B	SE B	β	*t*	*p*	95*%* BCa confidence intervals for B	
								Lower	Upper
**Model 1**	**0.26**	22.16***							
Age			0.03	0.00	**0.43**	7.18	**0.001**	0.02	0.04
Trait anxiety			− 0.04	0.05	− 0.05	− 0.88	0.389	− 0.13	0.04
Residence (urban:0; rural:1)			0.06	0.12	0.03	0.45	0.634	− 0.019	0.30
Education (HS: 0; HE:1)			− 0.31	0.11	− 0.**16**	− 2.71	**0.007**	− 0.53	− 0.05
**Model 2**	**0.36**	14.55***							
Age			0.02	0.00	**0.39**	6.63	**0.001**	0.02	0.03
Trait anxiety			− 0.06	0.04	− 0.08	− 1.35	0.194	− 0.14	0.01
Residence (urban:0; rural:1)			0.03	0.10	0.02	0.28	0.755	− 0.19	0.26
Education (HS: 0; HE:1)			− 0.25	0.10	− 0.**13**	− 2.32	**0.016**	− 0.44	− 0.02
Perceived severity of cancer treatment			0.03	0.05	0.03	0.49	0.635	− 0.07	0.13
Perceived susceptibility			0.03	0.08	0.02	0.38	0.754	− 0.10	0.18
Self-efficacy despite lack of time			0.21	0.07	**0.24**	3.89	**0.006**	0.06	0.35
Self-efficacy despite embarrassment			0.03	0.05	0.04	0.57	0.628	− 0.88	0.12
Self-efficacy despite pain			− 0.05	0.06	− 0.07	− 0.94	0.371	− 0.16	0.06
Response efficacy			0.26	0.10	**0.16**	2.73	**0.012**	0.07	0.45
**Model 3**	**0.45**	16.66***							
Age			0.01	0.00	0.11	1.57	0.118	− 0.00	0.02
Trait anxiety			− 0.04	0.04	− 0.05	− 1.00	0.325	− 0.11	0.04
Residence (urban:0; rural:1)			0.01	0.09	0.01	0.12	0.888	− 0.16	0.20
Education (HS: 0; HE:1)			− 0.20	0.09	− **0.10**	− 2.00	**0.041**	− 0.39	− 0.01
Perceived severity of cancer treatment			0.03	0.05	0.03	0.65	0.514	− 0.06	0.12
Perceived susceptibility			− 0.05	0.08	− 0.04	− 0.72	0.523	− 0.19	0.07
Self-efficacy despite lack of time			0.11	0.07	0.13	2.08	0.112	− 0.03	0.26
Self-efficacy despite embarrassment			0.05	0.05	0.08	1.11	0.343	− 0.05	0.13
Self-efficacy despite pain			− 0.06	0.05	− 0.09	− 1.30	0.260	− 0.16	0.05
Response efficacy			0.21	0.10	**0.13**	2.35	**0.032**	0.04	0.37
Breast checks frequency (Never: 0; W/Y: 1)			0.04	0.09	0.02	0.46	0.645	− 0.13	0.23
Mammography frequency Never 0 versus Irregular 1			0.89	0.16	**0.49**	6.10	**0.001**	0.59	1.19
Never 0 versus Regular 1			0.16	0.16	0.06	1.09	0.302	− 0.16	0.46

Results based on 1000 samples BCa bootstrapping

Bold values indicate statistically significant results (*p* < 0.05)

HS, high school and less; HE, some form of higher education; W/Y, weekly to yearly

***p < 0.001

In order to test the influence of previous breast screening behaviors on intention to attend mammography, both frequency of breast self-examination and previous mammography attendance were included in step 3. Addition of past breast screening behaviors (breast checks frequency and mammography frequency) to the model significantly increased prediction and the regression model accounted for 45.4% of variance in intention to attend mammography (*F* (13, 232) = 16.66, *p* < 0.001). With all variables entered into the model, the significant predictors of intention were mammography frequency (regular as compared to never attending) (β = 0.49, *p* = 0.001), response efficacy (β = 0.13, *p* = 0.032), and education level (β =  − 0.10, *p* = 0.041).

## Discussion

To our knowledge, this is the first quantitative study in the Norwegian context that examined associations between psychological factors and avoidance of breast cancer screening, and intention to attend mammography screening. The main finding was that the factors predicting defensive avoidance of breast cancer screening and intention to attend mammography screening were not the same, indicating that intention to attend screening may not be just the opposite of screening avoidance. The findings showed that defensive avoidance of breast cancer screening was associated with lower perceived susceptibility to breast cancer, lower response efficacy of mammography screening, higher breast cancer fear and checking breasts for lumps, while intention to attend mammography within the next two years was associated with higher response efficacy of mammography screening, having a lower educational level, and regular previous mammography attendance compared to never attending.

### Defensive avoidance of breast cancer screening

Following the propositions of the EPPM, we hypothesized that women with higher perceived threat and lower perceived efficacy would report higher defensive avoidance. Our findings, however, showed that women in our sample that perceived themselves as being less susceptible to breast cancer were more likely to hold defensive avoidance beliefs regarding breast cancer screening. While high perceived threat is reported as part of the fear control process within EPPM research [46], the majority of the EPPM studies use intervention designs that involve fear appeal messages and measure effects of fear appeals rather than existing perceptions. Thus, their results may differ from those obtained in cross-sectional studies. A study using cross-sectional design to examine the EPPM constructs got findings similar to ours, reporting the most defensive reactions among participants with low perceptions of threat and efficacy [47]. A potential explanation suggested by the researchers was that these low perceptions were a consequence of defensive reaction to some earlier fear appeal message that was highly threatening but conveyed too little efficacy and thus resulted in fear control response to minimize the perceptions of threat [47]. However, it is possible that our results indicate that women with low susceptibility to breast cancer in our sample think of breast cancer screening as not relevant. Stephenson and Witte [17] emphasized that defensive avoidance may be a hard construct to measure due to its ambiguity. They argued that after the participants of an intervention study were exposed to a fear appeal message, it was difficult to differentiate whether their responses to questions assessing defensive avoidance meant that they engaged in maladaptive coping as a result of a threatening message or simply ignored that message [17]. This corresponds with the previous studies on non-attendance of cervical cancer screening, which reported that women with low susceptibility to cervical cancer and women who felt healthy and had no symptoms were more likely to be non-attenders [48, 49], as were women who considered screening not relevant or not a priority [49].

We found that response efficacy of mammography screening was a significant negative predictor of defensive avoidance, indicating that women that do not consider mammography effective in averting breast cancer threat and making them feel safer may be less motivated to take part in it. This finding is consistent with prior fear appeal studies which showed that fear appeal messages with low efficacy generally led to greater fear control responses [46].

Our results showed that breast cancer fear was positively associated with defensive avoidance of breast cancer screening, meaning that women who worry a lot about getting breast cancer and are afraid to find a lump during screening may engage in defensive avoidance to rationalize why they do not need to attend screening, and thus reduce their anxiety. These findings are in line with previous research on breast cancer fear. Lagerlund, Hedin [8] found that those reporting that mammography would make them worry more about cancer were less likely to attend the screening. Similarly, Rippetoe and Rogers [50] reported that fear was directly related to defensive avoidance of performing breast self-examination.

Breast checks frequency was a significant negative predictor of defensive avoidance, meaning that those women who checked their breasts at least occasionally were less likely to have defensive avoidance beliefs regarding breast cancer screening. While this finding is contrary to Johansson and Berterö [51] and Lagerlund, Widmark [9] who reported that women practicing breast self-examination were more likely to not participate in the screening program, a possible explanation for our finding may be that our questions on defensive avoidance referred generally to breast cancer screening rather than just mammography, so women that check their breasts somewhat regularly may see it as protective against breast cancer.

Perceived severity of cancer treatment, self-efficacy despite lack of time, and mammography frequency were all significantly associated with defensive avoidance in the bivariate analysis, but not in the multivariate analysis. Perceived severity of cancer treatment positively correlated with breast cancer fear, while self-efficacy despite lack of time positively correlated with response efficacy of mammography screening. It is possible that a shared variance between the variables affected their ability to uniquely predict defensive avoidance. Mammography frequency was not a significant predictor of defensive avoidance even when entered into the model separately from breast checks frequency. This finding, therefore, could not be explained by the shared variance and needs to be examined further.

Finally, while it was observed in prior research that fewer immigrant women attend mammography screening in Norway [11], ethnicity was not a significant predictor of defensive avoidance of breast cancer screening in the multiple regression analysis. The bivariate results, however, showed that women with migration background reported higher mean defensive avoidance than did ethnic Norwegian women. Ethnic differences in defensive avoidance should be investigated further. A larger sample of women with migration background could have yielded different results and therefore should be recruited in future studies.

### Intention to attend mammography screening within the next two years

We hypothesized that women with higher perceived threat and higher perceived efficacy would report higher intention to attend mammography. In line with this, our findings showed that women who felt more confident that they could attend mammography despite having little time were more likely to express intention to attend the screening within the next two years. Further, those women who considered mammography to be effective in reducing breast cancer threat and making them feel safer were also more likely to report intention to attend screening. These findings are consistent with prior research. Higher response efficacy and higher self-efficacy were consistently found to be associated with recommended response to health threat for a number of health behaviors in the fear appeals research [18, 19, 34, 46]. Further, when it comes to breast cancer screening specifically, self-efficacy and response efficacy were significant predictors of breast screening behaviors in the studies that utilized other social cognition theories [e.g. 23,25,46]. In the Norwegian context, participants in a qualitative study of mammography experiences stated that being busy with their daily life was one of the main reasons to postpone mammography attendance [14]. This further supports our finding that women would be more likely to report intention to attend mammography if they scored higher on self-efficacy to attend mammography despite lack of time. Finally, Solbjør, Skolbekken [15] stated that Norwegian women who regularly attended mammography screening saw it as the only option to protect against breast cancer, thus indicating high response efficacy perceptions.

Contrary to our hypothesis, perceived susceptibility to breast cancer was not associated with intention to attend mammography in neither bivariate nor multivariate analysis. In previous studies, perceived susceptibility was not consistently associated with breast cancer screening adherence, showing significant associations in some studies [23–25], but not other [52, 53]. Similarly, in Norway, qualitative studies reported women having generally low perceived susceptibility of breast cancer, which did not change over the years of repeated mammography attendance [14, 15]. This may indicate that perceived susceptibility is of a lesser importance for women’s intention to get a mammogram when mammography screening is publicly available.

Having high school education or less (compared to having some form of higher education) was a significant positive predictor of intention to attend mammography. A number of earlier studies have found that women with higher education were less likely to take part in the national screening programs, with researchers suggesting that women with higher education may have been using private mammography services [e.g. 54–56]. However, a study in Denmark reported that women who did not participate in organized mammography screening, did not seek private mammography services either [57]. Jensen, Pedersen [56] further found that in a Danish sample, higher levels of education were associated with non-attendance, possibly as a result of making an informed choice not to attend screening after evaluating pros and cons of mammography screening.

Finally, we found that women, who have reported attending mammography once a year, or once every two years, were more likely to express intention to attend mammography screening compared to those who reported that they never attended mammography. Irregular attendance compared to never attending was not a significant predictor of intention. Moreover, after mammography frequency was added into the regression model, self-efficacy to attend mammography despite lack of time stopped being a significant predictor of intention, indicating stronger influence of regular mammography attendance on intention than self-efficacy. One possible explanation for this is that regular attendance of the screening program creates a habit and, therefore, some other psychological factors may become less salient. Accordingly, Solbjør, Skolbekken [15] reported that those women who consistently attended mammography between 2003 and 2009 perceived screening as a routine procedure that they did not question.

The findings in the current study provide support for the use of EPPM as a theoretical framework for studying breast cancer screening attendance and non-attendance. Our findings suggest that when designing health promotion programs to increase mammography screening attendance it is important to focus not just on predictors of screening attendance but also on predictors of screening avoidance. Our results show that the mechanism of defensive avoidance of breast cancer screening is complex. While a threatening message may increase perceived susceptibility to breast cancer which should lower defensive avoidance response, it may also increase fear which is positively associated with defensive avoidance. EPPM studies with intervention designs show that high threat + high efficacy messages distributed in health promotion campaigns are most effective to move individuals from the fear control to the danger control group [17, 46]. This could be an appropriate strategy in the case of women that are high on defensive avoidance in our sample. Moreover, designing campaign messages that would emphasize the effectiveness of mammography screening may be especially useful, as response efficacy was the only psychological predictor that was significant for both defensive avoidance and intention. Finally, our findings suggest that emphasizing regular use of the mammography screening program, thus making it a routine procedure for women, would be an effective strategy to ensure future consistent attendance.

The strengths of this study include focus on both intention to attend mammography screening and breast cancer screening avoidance. Further, the project recruited participants from different parts of Norway including the capital city and its suburbs, provincial towns and rural areas and had a fairly large sample. Finally, only a few studies have applied the EPPM in breast cancer screening research, thus our findings contribute to a better understanding of how the EPPM constructs predict breast cancer screening-related behaviors. The study also has some limitations. The cross-sectional design of the study does not allow drawing any conclusions on causality and directionality of the relationships or measuring future attendance of the mammography screening. Further, the study used questions taken from previously validated scales, but the scales were not used in their full versions. While construct validity for each sub-scale was assessed and they showed satisfactory reliability, it is possible that some constructs may not have been fully captured. Convenience sampling and recruitment from mammography centers may have affected the reported mammography rates. Furthermore, due to being an addition to the larger project that was not related to mammography screening, the study recruited a younger sample than is typically studied in mammography screening research: almost half of the women in our sample were below the age of 50. The response rate in the study was about 30%; therefore, there may be a self-selection bias. Thus, the findings of the study may not be generalizable to the general population. Finally, although the measures used in this study included some factors that may influence defensive avoidance and attendance of mammography screening, such as pain and discomfort, there are other possible factors that are not included. Factors that may influence screening decisions are availability of information on the pros and cons of mammography screening, recommendations from general practitioners, and communication with health personnel at the mammography centers.

Future research may benefit by investigating contextual factors that may affect screening participation. For example, Solbjør, Skolbekken [15] reported in their qualitative study that trust in the Norwegian healthcare system was an important aspect of mammography attendance.

## Conclusions

The present study used the Extended Parallel Process Model as a theoretical framework to examine the influences of psychological predictors on defensive avoidance of breast cancer screening and intention to attend mammography. The findings showed that defensive avoidance of breast cancer screening and intention to attend mammography were not influenced by the same psychological factors and are conceptually different. Thus, we argue that rather than treating avoidance of screening as the opposite of screening attendance, future health promotion campaigns should take into account that they are targeting two different health behaviors and need to consider not only the psychological factors that influence women’s decision to attend the screening, but also factors that contribute to women’s decision to avoid it.

## Supplementary Information

**Additional file 1: Appendix 1.** Full questionnaire used in the data collection.

## Data Availability

The datasets used and/or analysed during the current study are available from the corresponding author on reasonable request.

## Abbreviations

BSSS: The Breast Size Satisfaction Survey
EPPM: Extended Parallel Process Model
NBCSP: Norwegian Breast Cancer Screening Program
