# Supplementary material for: Psychological predictors of intention and avoidance of attending organized mammography screening in Norway: applying the Extended Parallel Process Model
Source: BMC Womens Health. 2021 Feb 15;21:67. doi: 10.1186/s12905-021-01201-y (PMC7885347; doi:10.1186/s12905-021-01201-y)
Supplement: Supplementary file 1 — Additional file 1: Appendix 1. Full questionnaire used in the data collection. [file 12905_2021_1201_MOESM1_ESM.pdf]

Psychological predictors of intention and avoidance of attending organized mammography screening in Norway - applying the Extended Parallel Process Model

Anna Ivanova[1*]

Ingela Lundin Kvalem[1]

[1] Department of Psychology, University of Oslo

[*]Correspondence: anna.ivanova@psykologi.uio.no

Department of Psychology,

University of Oslo,

PB 1094, Blindern,

N-0317 Oslo, Norway

Appendix 1. Full questionnaire used in the data collection for the BSSS project [1] and the present study

☐ ***I have read the information sheet and know that the answers are anonymous***

We are asking these questions to find out more about breast size satisfaction across countries. It is not a test. We are interested in your thoughts and beliefs, so please answer the questions as honestly as you can. All your answers will be treated as strictly confidential and the questionnaire itself is anonymous.

The first few questions are about you.

1. What is your age? ________________

2. What is your height? ________________ 3. What is your weight? ________________

4. In the country of your residence, which of the following best describes your ethnic/racial affiliation?

☐ Ethnic/racial majority ☐ Ethnic/racial minority ☐ Not sure

5. Which of the following best describes your current place of residence?

☐ Capital city ☐ Capital city suburbs ☐ Provincial city (more than 100,000 residents)
☐ Provincial town (more than 10,000 residents) ☐ Rural areas

6. Which of the following best describes your education?
☐ No formal education
☐ Primary education
☐ Secondary education
☐ Still in full-time education
☐ Undergraduate degree
☐ Postgraduate degree
☐ Other

7. Compared to others of your own age in your country of residence, how financially secure do you feel?

☐ Less secure ☐ Same ☐ More secure

8. Have you ever had breast cancer? ☐ Yes ☐ No ☐ Don't know

These next questions are about finding changes in your breasts.

9. How often do you check your breasts?
☐ Rarely or never
☐ At least once every 6 months
☐ At least once a month
☐ At least once a week

10. Are you confident you would notice a change in your breasts?
☐ Not at all confident
☐ Slightly confident
☐ Fairly confident
☐ Very confident

11. Have you ever been to see a doctor about a change you have noticed in one of your breasts?

☐ Yes ☐ No ☐ Never noticed a change in one of my breasts

12. If you found a change in your breasts, how soon would you contact your doctor? ____________________

____ (please write in your response)

13. This question is about who you think is most likely to get breast cancer. Tick one box only. In the next year, who is most likely to get breast cancer?

☐ A 30-year-old woman
☐ A 50-year-old woman
☐ A 70-year-old woman
☐ A woman of any age

14. This question is about how many women you think will develop breast cancer in their lifetime. Please imagine groups of 3, 9, 100, and 1000 women, and tick one box only. How many women will develop breast cancer in their lifetime?

☐ 1 in 3 women ☐ 1 in 9 women ☐ 1 in 100 women ☐ 1 in 1000 women ☐ Don't know

The next questions ask about your attitudes and feelings about yourself. Please circle an appropriate answer using the 1-7 scale.

	Extremely dissatisfied			*Neutral*			*Extremely satisfied*
15. How dissatisfied or satisfied are you with your physical appearance?	1	2	3	4	5	6	7
16. How dissatisfied or satisfied are you with your weight?	1	2	3	4	5	6	7

The next questions ask about how you view yourself. Please note that the response options have changed.

	Strongly disagree				*Strongly agree*
17. I see myself as open to new experiences.	1	2	3	4	5
18. I see myself as dependable and self-disciplined.	1	2	3	4	5
19. I see myself as extroverted and enthusiastic.	1	2	3	4	5
20. I see myself as warm and sympathetic to others.	1	2	3	4	5
21. I see myself as anxious and easily upset.	1	2	3	4	5

22. Taking all things together, how would you say things are these days?

☐ Not too happy ☐ Pretty happy ☐ Very happy

This question is about your self-esteem. Please rate how true the following statement is about you.

	Not very true of me						*Very true of me*
23. I have high self-esteem.	1	2	3	4	5	6	7

The next few questions are about your use of different types of media.

In your lifetime, how much have you…	*Less than once a month*	*Once or twice a month*	*Once a week*	*Several times a week*	*Every day*
24. Watched Western/US/EU television shows?	1	2	3	4	5
25. Watched Western/US/EU movies?	1	2	3	4	5
26. Read Western/US/EU magazines?	1	2	3	4	5
27. Used Western/US/EU internet sites?	1	2	3	4	5
28. Watched Norwegian television shows?	1	2	3	4	5
29. Watched Norwegian movies?	1	2	3	4	5
30. Read Norwegian magazines?	1	2	3	4	5
31. Used Norwegian internet sites?	1	2	3	4	5

Finally, please use the images on the next page to answer the following questions.

32. Which figure most closely matches your **current** breast size? ____________________

33. Which is the breast size that you would **most like to possess**? ____________________

34. Which figure do you think most closely matches the **average breast size of women** in your country? ____________________

35. Which figure do you think most closely represents the breast size that **men in your country** find most attractive? ____________________

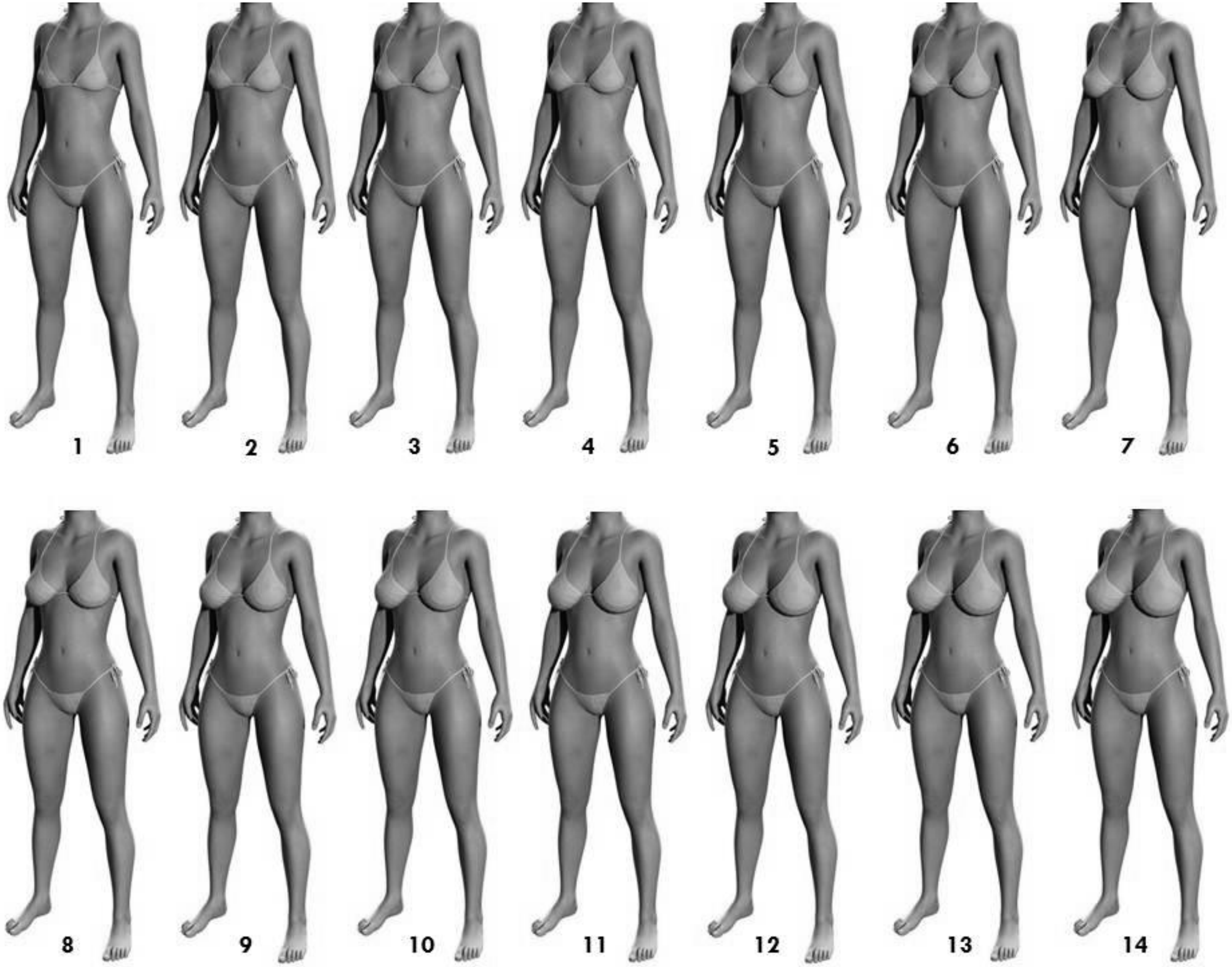

1
2
3
4
5
6
7
8
9
10
11
12
13
14

Now there are some more questions about breast cancer and breast examination

Mammogram = breasts X-ray

36. For someone of my age my health in general is: *Check the box that applies to you*	**Excellent**	**Good**	**Average**	**Not that good**	**Poor**
37. I believe my chance of developing breast cancer in my lifetime is: *Check the box that applies to you*	**Very low**	**Moderately low**	**Neither high or low**	**Moderately high**	**Very high**
38. My chance of developing breast cancer compared to the average woman of my age is: *Check the box that applies to you*	**Much lower**	**Somewhat lower**	**About the same**	**Somewhat higher**	**Much higher**

39. Have you ever known anyone personally who has breast cancer?	☐ Yes	☐ No
If yes, please circle who	☐ immediate family member	☐ distant relative
	☐ a friend	☐ an acquaintance

40. Can a person have a breast cancer but not have any symptoms?	☐ Yes	☐ No	☐ I don't know

Check the box for each of the questions	**Strongly agree**	**Agree**	**Neither agree or disagree**	**Disagree**	**Strongly disagree**
41. I frequently do things to improve my health					
42. I receive enough understandable information from my doctor to make good decisions about my health					
43. I believe that breast cancer treatment is hard to endure					
44. I believe that chances of survival from breast cancer are low					
45. I worry a lot about developing breast cancer					
46. Examining my breasts or getting mammography would make me worry more about cancer					
47. I am afraid of finding a lump in my breast during self-examination or mammography					

48. Have you ever had a mammogram?	☐ Yes		☐ No (go to question 51)	☐ I don't know	
49. How often do you go to get a mammogram?	Once a year	Once in two years	Less than once in two years	Only if I get symptoms	Never
50. How did you get a mammogram the last time?	☐ Through the screening program			☐ My own initiative	

Check the box for each of the questions	**Yes, definitely**	**Yes, probably**	**Probably not**	**No**
51. I intend to perform breast self-examination **in the next month**.				
52. I intend to attend mammography **within the next two years.**				

Check the box for each of the questions	**Strongly agree**	**Agree**	**Neither agree or disagree**	**Disagree**	**Strongly disagree**
**About breast self-examination**					
53. I am certain I can remember to do breast self-examination within the next month					
54. I am embarrassed to touch my breasts					
55. I do not have any symptoms so I don't need to do breast self-examination					
56. I don't know how to do breast self-examination					
57. I think it is important do breast self-examination					
58. Performing breast self-examination would make me feel safe					
**About mammography**					
59. I am certain I can attend mammography even though …					
… I have little time					
… I am embarrassed to expose my body to others					
… mammography may be painful					
60. Exposing my body to others is forbidden by my religion/culture					
61. Getting a mammogram is bad for health					
62. I do not have any symptoms so I don't need to do a mammogram					
63. I think it is important to do a mammogram					
64. Getting a mammogram would make me feel safe					
**About checking your breasts**					
65. If I examined my breasts or got a mammogram regularly, it is more likely to detect cancer early					
66. If I found a lump in my breast, it would not really matter, because by then it's too late anyway					
67. Detecting breast cancer early would make treatment more successful					
68. There are so many things that could happen to me that it is pointless to think about something like breast cancer					
69. My health is too good at present to even be thinking I might get breast cancer					

70. Are you born in Norway? ☐ Yes ☐ No If not, what country?___________________________

71. Was your mother born in Norway? ☐ Yes ☐ No If not, what country?___________________________

72. Was your father born in Norway? ☐ Yes ☐ No If not, what country?___________________________

If you have any comments or there is something you want to tell us that we forgot to ask about, please write it here!

Thank you for your help!

References:

1. Swami V, Tran US, Barron D, Afhami R, Aimé A, Almenara CA, et al. The Breast Size Satisfaction Survey (BSSS): Breast size dissatisfaction and its antecedents and outcomes in women from 40 nations. Body Image. 2020;32:199-217.
